# Divergence of feeding channels within the soil food web determined by ecosystem type

**DOI:** 10.1002/ece3.905

**Published:** 2013-12-04

**Authors:** Felicity V Crotty, Rod P Blackshaw, Sina M Adl, Richard Inger, Philip J Murray

**Affiliations:** 1 Sustainable Soil and Grassland Systems, Rothamsted ResearchNorth Wyke, Okehampton, EX20 2SB, U.K; 2 Centre for Agricultural and Rural Sustainability, Plymouth UniversityDrake Circus, Plymouth, PL4 8AA, U.K; 3 Department of Soil Science, University of Saskatchewan51 Campus Drive, Saskatoon, S7N 5A8, Canada; 4 Institute of Biological, Environmental and Rural Sciences, Aberystwyth UniversityGogerddan, Aberystwyth, SY23 3EE, U.K; 5 Biosciences, Daphne du Maurier Building, University of ExeterCornwall Campus, Penryn, TR10 9EZ, U.K

**Keywords:** Community structure, decomposers, energy channels, food webs, soil ecology, stable isotopes.

## Abstract

Understanding trophic linkages within the soil food web (SFW) is hampered by its opacity, diversity, and limited niche adaptation. We need to expand our insight between the feeding guilds of fauna and not just count biodiversity. The soil fauna drive nutrient cycling and play a pivotal, but little understood role within both the carbon (C) and nitrogen (N) cycles that may be ecosystem dependent. Here, we define the structure of the SFW in two habitats (grassland and woodland) on the same soil type and test the hypothesis that land management would alter the SFW in these habitats. To do this, we census the community structure and use stable isotope analysis to establish the pathway of C and N through each trophic level within the ecosystems. Stable isotope ratios of C and N from all invertebrates were used as a proxy for trophic niche, and community-wide metrics were obtained. Our empirically derived C/N ratios differed from those previously reported, diverging from model predictions of global C and N cycling, which was unexpected. An assessment of the relative response of the different functional groups to the change from agricultural grassland to woodland was performed. This showed that abundance of herbivores, microbivores, and micropredators were stimulated, while omnivores and macropredators were inhibited in the grassland. Differences between stable isotope ratios and community-wide metrics, highlighted habitats with similar taxa had different SFWs, using different basal resources, either driven by root or litter derived resources. Overall, we conclude that plant type can act as a top-down driver of community functioning and that differing land management can impact on the whole SFW.

## Introduction

It is of critical importance that we begin to understand food webs in different environments and not just the biodiversity. Assessing which function an organism performs is far more important than merely counting them. Food webs provide a quantitative framework to combine community ecology with ecosystem ecology and unify the study of biodiversity and ecosystem function (Thompson et al. 2012). This article represents our assessment of the soil mesofauna food web and how it functions under differing land management. Within all soil food webs (SFW), there is a perceived paradox between the large diversity of organisms (densely packed within space and time) and the level of feeding specialization. There is a misconception that there are not enough individual niches for the number of different species found within the soil (Coleman 2008). The factors responsible for this high diversity of soil animals are not fully understood (Maraun et al. 2011). Soil biota have a large impact on nutrient cycling both directly (e.g., comminution, litter decomposition (Ponge 1991), and root herbivory (Murray and Clements 1998; Treonis et al. 2005)), and indirectly (e.g., burrowing, casting and fecal deposition changing soil porosity and aggregate formation (Davidson and Grieve 2006)). Litter decomposition is determined by interactions between resource (plant) quality and the consumers (decomposers), which are both controlled by the environment (climatic and soil conditions) (Makkonen et al. 2012). We still do not know how vital each individual species are, or the level of influence a change in plant species can have on a soil fauna community at either the local or global scale.

There are large differences in plant assemblage between woodlands and grasslands. It is known that soil biota can affect plant succession and competition (Bonkowski and Roy 2012). In woodlands, the additional understory forbs as well as the reduction in light at ground level due to the canopy increase the potential niches, favouring surface dwellers that prefer low light levels and overall increases the spatial variability (Berg and Bengtsson 2007). Although grasslands are considered to be one of the most species rich habitats in the world (Wilson et al. 2012), they are also continuously foliated, providing a year round food source. There is a lack of detailed grassland food web structure (Kohzu et al. 2009), which makes it harder to relate different habitat types to each other.

Stable isotope ratio analysis is one method that can be used to assess the feeding strategies of the soil faunal community. Studies on the whole SFW have shown that the food chains appear to be relatively short, with decomposers separated from predators (Ponsard and Arditi 2000). Individual species analysis has, however, shown a continuum of stable isotope ratios (Chahartaghi et al. 2005). From this continuum, individual feeding niches can be inferred. Most of the studies to date have investigated just one habitat or habitats of differing humus or soil type. Few studies have compared differences between habitats or land management of the same soil type, using the same taxonomic parameters for separation. Many studies focus on the dominance of the bacterial or fungal energy channel and imply that differences are due to management practice, plant type, and soil characteristics (acidity, organic matter content) (Strickland and Rousk 2010). Within our research, soil characteristics are controlled, with differences having been accrued through a single management change (grassland to woodland) approximately 25 years ago.

The key issue that is currently poorly understood is how different trophic levels within the SFW are affected by plant type and management. Here, our investigation utilizes a novel opportunity focusing on two ecosystems that were originally the same, but for a conversion, in management. These two ecosystems have the same soil type, which acts as a control, reducing the number of variables between these two systems, where differences between trophic groups will solely be due to plant and management change. We wanted to define the trophic structure of the food web using stable isotope ratios of nitrogen (N) and carbon (C) and to determine whether the same organisms have different functions within the different habitats. Finally, we wanted to assess whether there were differences between the soil fauna for C, N, and C/N between habitats compared with historical data. We addressed these aims through stable isotope analysis of the SFW in permanent grassland and nearby woodland both derived from the same grassland and soil type.

## Material and Methods

### Soil preparation and sampling

Intact soil cores (10 cm ∅, 10 cm deep, *n* = 6 per habitat) were taken from permanent agricultural grassland (50°46′55″N, 3°55′1″W) and a willow (*Salix* sp.) woodland site (50°46′16″N, 3°54′22″W) both located at Rothamsted Research (North Wyke). Both sites were of the same soil type Hallsworth series (Harrod and Hogan 2008), which is a clayey pelo-stagnogley soil in head from clay shale, located mainly under low-lying slopes. The grassland site had received no inorganic-N input for over 25 years but was annually grazed by cattle. The willow woodland was planted approximately 25 years ago. Details of the soil characteristics and weather conditions at the sites can be found in Crotty et al. (2012).

The cores were removed by driving individual polypropylene sleeves (11.4 cm external diameter, 11 cm deep) into the soil, to retain the entire faunal assemblage within the core and leaving the flora intact on the core surface. Each core was stored for 48 h within an individual Sun-bag (Sigma-Aldrich, MO, St Louis), in a controlled environment chamber, (12/12 h light/dark period and 18/13°C temperature cycling, 40% relative humidity), until the extraction of invertebrates. Prior to invertebrate extraction from each core, the vegetation (grass/under-canopy forbs) was cut to ground level and oven-dried for 24 h at 105°C and finely ground before analysis by mass spectrometry. Dead plant material (grass and willow senesced leaf litter) was removed from the two sites for bulk stable isotope analysis and prepared following the same method as above for other plant material.

The core was removed from the plastic sleeve, and a vertical slice (approximately 150 g) was removed and homogenized. Of this homogenized sample, 100 g was used for nematode extractions, and 50 g for dry weight and bulk isotope analysis. Nematode extractions were performed following the methods of Crotty et al. (2011) adapted from (Whitehead and Hemming 1965). Soil was oven-dried for 24 h at 105°C to assess dry weights and ground prior to analysis by mass spectrometry.

### Meso- and macrofauna sampling

The remainder of the core was placed on a Tullgren funnel system (mesh 5 mm) (Burkard Manufacturing Co. Ltd, Rickmansworth, UK) for 10 days. The invertebrates were collected in saturated salt solution to maintain isotopic composition. Invertebrate groups were identified and separated, under a microscope, prior to drying and analysis. Invertebrates were transferred to tin capsules and dried at 65°C for 48 h prior to continuous flow stable isotope ratio mass spectrometry. Invertebrates were separated into the four main Collembola orders – Entomobryomorpha, Poduromorpha, Neelipleona, and Symphypleona; and the Acari – Mesostigmata, Prostigmata, Oribatida, and Astigmata. Other invertebrates were separated to order, except the Coleoptera which were separated to family; Diptera were sorted to order apart from Tipulidae larvae which were analyzed separately. All fauna were sampled with their gut contents intact (with the exception of earthworms, Tipulidae larvae, and slugs, whose gut track and content were removed through dissection).

### Stable isotope analysis

Sample material of invertebrates, soils, and foliage were analyzed for total N and C contents and the ^15^N/^14^N and ^13^C/^12^C isotope ratios, along with analytical quality control samples. The isotope concentrations were determined using a Flash EA 1112 Series Elemental Analyser connected via a Conflo III interface to a DeltaPlus XP isotope ratio mass spectrometer (all Thermo Finnigan, Bremen, Germany). The precision range was 20–300 *μ*g C and 15–150 *μ*g N (low C run) and 400–4000 *μ*g C and 30–900 *μ*g N (normal C run), with an analytical precision of ±0.29 ‰ for *δ*^13^C and ±0.0002 atom% for ^15^N. Where samples of individual groups of invertebrates had too low a biomass for the precision range of the mass spectrometer, these samples were bulked between cores within the same habitat.

Stable isotopes at natural abundance are expressed using the *δ* notation with *δ*^13^C (‰) and *δ*^15^N (‰) calculated using the equation: *δ*^*n*^*E* (‰) = (*R*_sample_−*R*_standard_)/*R*_standard_ where *E* is the element (C or N), *n* is the weight of the heavier (rarer) isotope, and *R* is the ratio of the heavy to light isotopes (Tiunov 2007). *R*_sample_ and *R*_standard_ represent the ^13^C/^12^C or ^15^N/^14^N ratios of the sample and standard, respectively. For ^15^N, atmospheric N_2_ served as the primary standard, and for ^13^C, it was Vienna Pee Dee Belemnite (VPDB). The standards for C and N *R*_standard_ are equal to 1.1237 × 10^−2^ and 3.6764 × 10^−3^ atom% respectively.

Bearhop et al. (2004) postulated that stable isotope analysis can identify trophic niches within an ecosystem and Layman et al. (2007), developed methods to test for these community-wide metric values. The differences between the communities as a whole was assessed through “Stable Isotope Bayesian Ellipses in R” (SIBER) (Jackson et al. 2011), using the *δ*^15^N and *δ*^13^C results for both habitats for all soil fauna for community-wide metrics.

### Statistical analysis

All data were analyzed using the statistical package GenStat (GenStat 13, VSN International Ltd., Hemel Hempstead, UK), unless otherwise stated. All population data were normalized by transformation [log_10_ (*x* + 1)] prior to analysis. All data were analyzed by a general regression analysis as well as a Student's *t*-test (unpaired two sample, two sided). The Student's *t*-tests were used to compare isotopic composition between fauna in each habitat, and also to compare C/N ratios described in the literature to our results. An analysis of variance (ANOVA), with habitat as the main factor, was applied to determine differences in organism numbers and delta values within the different habitats, as well as differences in community-wide metrics (which were generated using the statistical program R (R Development Core Team 2008)).

When analyzing functional groups, ANOVA was also used combined with Fisher's protected least significant difference (FPLSD) test. An assessment of the relative response of the different functional groups to the change from agricultural grassland to woodland was performed using the equation *V* = [2*M*_gr_/(*M*_gr_ + *M*_w_)]−1 based on the equation by Wardle (1995) where *M*_gr_ and *M*_w_ = abundance of organisms in each functional group in either the agricultural grassland (*M*_gr_) or woodland (*M*_w_). The index ranges from −1 (functional groups extremely inhibited by agricultural grassland) to +1 (functional groups extremely stimulated by agricultural grassland), with 0 indicating relatively equal abundances under both systems. All data presented as mean ± standard error, unless otherwise stated. As isotope signatures represent soil fauna from two different habitats, after initial analysis, results were normalized using the methods of Erdmann et al. (2007), by setting the stable isotope signatures of the soil to zero and calibrating all other sample signatures accordingly.

## Results

### Soil and vegetation characteristics

The grassland soil had a significantly higher C and N content than the woodland (%C: *F*_1,10_ = 36.81; *P* < 0.001 and %N: *F*_1,10_ = 82.21; *P* < 0.001; Table S1), but C/N ratios and bulk densities were not significantly different between habitats. The *δ*^13^C and *δ*^15^N values were significantly different though, with those of the grassland being lower compared with the woodland (*δ*^13^C *F*_1,10_ = 86.10; *P* < 0.001 and *δ*^15^N *F*_1,10_ = 43.09; *P* < 0.001; Table S1, other soil characters (Crotty et al. (2012)).

Total C and N content of the vegetation in the two habitats reflected that of the soil, with those of the grassland being significantly greater (%C: *F*_1,8_ = 9.82; *P* = 0.014 and %N: *F*_1,8_ = 35.50; *P* < 0.001; Table S1). However, the vegetation *δ*^13^C and *δ*^15^N signatures were very similar between the two habitats, (Table S1). Analysis of the plant litter showed the grassland to have a significantly lower C and N content compared with the woodland (C *F*_1,10_ = 778.82; *P* < 0.001 and N *F*_1,10_ = 102.23; *P* < 0.001 Table S1), although the C/N ratios were not different. The *δ*^13^C of the litter was not different between habitats, −30.0‰ (±0.04) in the grassland, while being −30.1‰ (±0.06) in the woodland. However, the *δ*^15^N signatures were significantly lower in the grassland habitat, −0.4‰ (±0.10) compared with 2.1‰ (±0.05) (*F*_1,10_ = 563.02; *P* < 0.001).

Living plant material had a significantly lower C content (*F*_1,18_ = 98.11; *P* < 0.001) and C/N ratio (*F*_1,18_ = 63.25; *P* < 0.001) compared with the dead material (Table S1). Furthermore, the *δ*^13^C and *δ*^15^N signatures were also significantly lower in both habitats for the plant litter, in comparison with the living material (*F*_1,18_ = 10.36; *P* = 0.005 and *F*_1,18_ = 39.82; *P* < 0.001 respectively).

### Community composition within the soil food web

There were significant differences between the population numbers and biomass for many of the macro- and mesofauna taxa (Table S2), although these were not consistent between habitats. These variations represent divergence in community structures in the two habitats, indicating different functional food web interactions occurring.

There were few significant differences in the C and N content and C/N ratio for the soil invertebrates between the two habitats (Table 1). The only exceptions were aphids for C content (*F*_1,2_ = 428.88; *P* = 0.002) and C/N ratio (*F*_1,2_ = 77.12; *P* = 0.013), and Collembola Entomobryomorpha for N content (*F*_1,4_ = 8.42; *P* = 0.044), which were all higher in the grassland. The Poduromorpha had significantly higher %N (the only group to be higher in the woodland (*F*_1,2_ = 43.01; *P* = 0.022)).

**Table 1:** Analysis of C and N content of the soil fauna from the grassland and woodland habitats

	%C		%N		C:N ratio		Hunt C/N ratio *t*-test
				
	Grassland	Woodland	Grassland	Woodland	Grassland	Woodland	
Acari: Astigmata	21.5	16.9	3.5	3.4	6.1	5.0	−7.63; *P* = 0.005
Acari: Mesostigmata	39.5 (±1.36)	42.5 (±3.48)	9.7 (±0.73)	10.0 (±0.54)	2.9 (±1.47)	2.7 (±1.36)	−33.97; *P* < 0.001
Acari: Mesostigmata: Uropodidae	43.8 (±1.01)		8.6 (±0.46)		5.1 (±0.16)		−18.42; *P* = 0.035
Acari: Oribatida	40.4 (±1.22)	37.6 (±1.09)	7.7 (±0.12)	7.3 (±0.40)	5.2 (±0.14)	3.3 (±1.64)	−20.77; *P* < 0.001
Acari: Oribatida: Phthiracaridae		21.4 (±0.20)		2.9 (±0.04)		7.3 (±0.02)	−19.20; *P* = 0.033
Acari: Prostigmata	25.6 (±1.95)	30.0 (±4.19)	4.9 (±0.54)	5.8 (±1.07)	5.2 (±0.24)	5.2 (±0.22)	−15.22 *P* < 0.001
Aphids (Hemiptera: Aphidoidea)^1^,^2^	40.5 (±0.48)	26.5	4.8 (±0.19)	4.9	8.5 (±0.25)	5.4	
Chilopoda: Geophilomorpha		30.9 (±1.32)		5.0 (±0.88)		5.0 (±2.49)	
Coleoptera Larvae		21.4	6.6	4.9		4.4	
Coleoptera Larvae: Elateridae	29.2		6.6		4.4		
Coleoptera Larvae: Staphylinidae	15.0 (±0.38)	16.5	3.4 (±1.11)	3.6	4.4 (±2.22)	4.6	
Coleoptera: Carabidae		37.2		5.5		6.7	
Coleoptera: Ptiliidae		37.4		4.8		7.8	
Coleoptera: Staphylinidae	28.5	31.2 (±1.22)	4.5	4.9 (±0.28)	6.4	6.4 (±0.45)	
Collembola: Entomobryomorpha^3^	45.0 (±0.45)	36.1 (±3.34)	7.2 (±0.42)	5.0 (±0.65)	6.3 (±0.32)	7.3 (±0.30)	−2.68; *P* = 0.075
Collembola: Neelipleona		16.1 (±5.49)		2.1 (±0.67)		7.7 (±0.15)	−2.07; *P* = 0.286
Collembola: Poduromorpha^2^	49.3 (±5.83)	48.7 (±1.53)	6.8 (±1.36)	4.0 (±0.36)	7.3 (±0.60)	12.3 (±0.71)	1.22; *P* = 0.309
Collembola: Symphypleona	16.7	15.2	2.8	3.1	5.9	4.9	−8.74; *P* = 0.003
Diplopoda: Julidae		24.3 (±4.44)		3.7 (±0.60)		6.5 (±0.13)	
Diplopoda: Polydesmidae		27.7 (±2.71)		4.4 (±0.41)		6.3 (±0.06)	
Diptera	27.5	23.2 (±1.36)	6.3	5.0 (±0.25)	4.4	4.7 (±0.12)	
Diptera Larvae	18.7 (±3.08)	14.0 (±3.11)	3.8 (±1.12)	3.7	5.8 (±1.63)	5.4	
Earthworm	30.5 (±7.36)	32.1 (±5.76)	6.8 (±1.92)	7.0 (±0.96)	4.6 (±0.20)	4.6 (±0.34)	
Enchytraeids	22.8	34.1	5.4	6.9	4.3	5.0	
Nematodes	11.2 (±0.13)	9.6 (±0.81)	1.2 (±0.16)	1.1 (±0.21)	9.7 (±1.61)	8.8 (±0.84)	−0.90; *P* = 0.411
Pseudoscorpion		23.5		5.2		4.5	
Snail		13.0		1.5		8.7	
Spider	34.8	38.2 (±6.40)	6.3	7.7 (±2.80)	5.6	5.4 (±1.11)	
Thrips	37.0		5.8		6.4		
Woodlice	15.1	16.5 (±1.64)	2.7	2.6 (±0.11)	5.7	4.3 (±2.17)	

Data presented as mean ± standard error (*n* = 3). Single-factor ANOVA indicating differences between habitats was not significant for the majority of invertebrates apart from those labeled. Student's *t*-test was performed to assess whether the invertebrates had different C/N ratios in comparison with Hunt et al. (1987), which has been used over the last 20 years for modeling soil fauna ecological interactions, where Acari and Collembola have a C/N ratio of 8, and Nematodes have a C/N ratio of 10; (df 1–5) habitats were combined for the analysis.

1 For %C – Aphids *F*_1,2_ = 427.128.88; *P* = 0.002.

2 For C:N ratio – Collembola: Poduromorpha *F*_1,2_ = 43.01; *P* = 0.022; and Aphids *F*_1,2_ = 77.12; *P* = 0.013.

3 For %N – Collembola Entomobryomorpha *F*_1,4_ = 8.42; *P* = 0.044.

Testing the C/N ratio of the Acari and Collembola found here, in relation to published data (C/N ratio of 8 as stated by Hunt et al. (1987)) found significant variation dependent on habitat, lineage, or superfamily. The Acari were found to have significantly lower ratios in both habitats (grassland 5.2 ± 0.19; woodland 5.3 ± 0.36) to the expected (*t* = −13.76; df_19_; *P* < 0.001). All the individual lineages (Mesostigmata, Astigmata, Oribatida, and Prostigmata) in both the grassland and the woodland also had significantly lower C/N ratios (Table 1), with the Mesostigmata being particularly low (>3 for both habitats). Collembola inhabiting the grassland were also significantly different (*t* = −4.50; df_5_; *P* = 0.006), with overall means being significantly lower in the grassland (6.6 ± 0.31), although the individual super-families were not, apart from the Symphypleona which also had a C/N ratio that was significantly lower than that stated by Hunt et al. (1987) in both habitats (Table 1). However, the C/N ratio of the Nematodes was not significantly different to the C/N ratio of 10 stated by Hunt et al. (1987).

### *δ*^13^C and *δ*^15^N signatures of the soil fauna

Prior to normalization for variation in soil isotopic signatures, an analysis of variance was performed for the *δ*^13^C and *δ*^15^N signatures of the soil fauna (Table 2; all *F* and *P* values can be seen in Table 2). There was variation between the two habitats, although there was a large overlap when plotted on the same graph (figure not shown). The *δ*^15^N signatures of many invertebrates were significantly different between habitats, including the Oribatida, Prostigmata, Staphylinidae larvae, Entomobryomorpha, and woodlice, all having higher *δ*^15^N signatures in the grassland (Table 2). However, the *δ*^15^N signature of soil was lower in the grassland than the woodland, opposite to expected signatures if habitat was solely affecting the results. The Poduromorpha were the only group which had significantly higher *δ*^15^N values in the woodland (Table 2).

**Table 2:** Average delta signatures for *δ* ^13^C and *δ* ^15^N for the soil fauna from the grassland and woodland habitats

		Grassland		Woodland		*F-*values	
				
	Abbreviation	*δ*^13^C	*δ*^15^N	*δ*^13^C	*δ*^15^N	*δ*^13^C	*δ*^15^N
Acari: Astigmata	aa	−26.23	5.46	−26.73	4.16		
Acari: Mesostigmata	am	−26.61 (±0.198)	9.73 (±0.659)	−26.55 (±0.047)	7.52 (±0.543)	0.15_1,2_	6.64
Acari: Mesostigmata: Uropodidae	amu	−26.69 (±0.204)	10.45 (±0.204)				
Acari: Oribatida	ao	−28.08 (±0.085)	5.92 (±0.370)	−27.08 (±0.119)	2.16 (±0.138)	62.95*_1,3_	90.71**
Acari: Oribatida: Damaeidae	aod			−24.32	5.52		
Acari: Oribatida: Phthiracaridae	aop			−22.94 (±0.029)	3.03 (±0.022)		
Acari: Prostigmata	ap	−27.63 (±0.656)	6.72 (±0.598)	−28.47 (±0.268)	4.20 (±0.345)	1.41	13.40*
Aphids (Hemiptera: Aphidoidea)	ha	−30.66 (±0.266)	2.56 (±0.623)	−33.28	4.32	48.46*_1,2_	3.97_1,2_
Chilopoda: Geophilomorpha	cg			−27.51 (±0.207)	7.54 (±0.973)		
Coleoptera Larvae	cl			−26.20	5.87		
Coleoptera Larvae: Elateridae	cle	−27.21	4.70				
Coleoptera Larvae: Staphylinidae	cls	−28.32 (±0.293)	6.42 (±0.221)	−26.17	4.28	40.60_1,2_	46.54*_1,2_
Coleoptera: Carabidae	ccb			−28.09	4.66		
Coleoptera: Ptiliidae	cpt			−28.15	2.99		
Coleoptera: Staphylinidae	cst	−28.87	4.62	−27.61 (±0.272)	5.93 (±0.677)	10.81_1,2_	1.88_1,2_
Collembola: Entomobryomorpha	ce	−29.08 (±0.412)	5.15 (±0.176)	−28.96 (±0.135)	1.18 (±0.900)	0.07	18.71*
Collembola: Neelipleona	cn			−27.25 (±0.306)	4.30 (±1.665)		
Collembola: Poduromorpha	cp	−28.15 (±0.440)	6.66 (±0.204)	−27.79 (±0.045)	9.23 (±0.687)	0.98_1,2_	19.26*_1,2_
Collembola: Symphypleona	csy		2.10	−27.64	−0.16		
Diplopoda: Julidae	dj	−28.56		−25.87 (±1.252)	1.89 (±0.278)		
Diplopoda: Polydesmidae	dp			−25.61 (±0.255)	4.25 (±0.814)		
Diptera	d	−27.62	7.23	−28.96 (±0.359)	10.44 (±0.875)	6.95_1,2_	6.70_1,2_
Diptera Larvae	dl	−27.27 (±0.603)	5.40 (±0.9777)	−32.66 (±3.517)	4.9	2.28	0.13_1,2_
Earthworm	ew	−28.26 (±0.188)	4.55 (±1.474)	−26.24 (±0.366)	3.89 (±0.128)	21.04*_1,3_	0.43_1,3_
Enchytraeids	ec	−26.92	4.99	−27.14	3.15		
Nematodes	n	−26.75(±1.018)	7.44 (±0.331)	−27.65 (±0.279)	3.29 (±2.076)	0.72	4.09
Pseudoscorpion	ps			−27.07	3.90		
Snail	sn			−21.02	0.82		
Spider	sp	−28.40	6.90	−26.83 (±0.596)	8.78 (±1.066)	5.23_1,2_	2.33_1,2_
Thrips	t	−29.50	3.75				
Woodlice	w	−29.10	3.43	−25.86 (±0.847)	3.16 (±0.033)	0.06_1,2_	33.37*_1,2_

**Figure 1:**
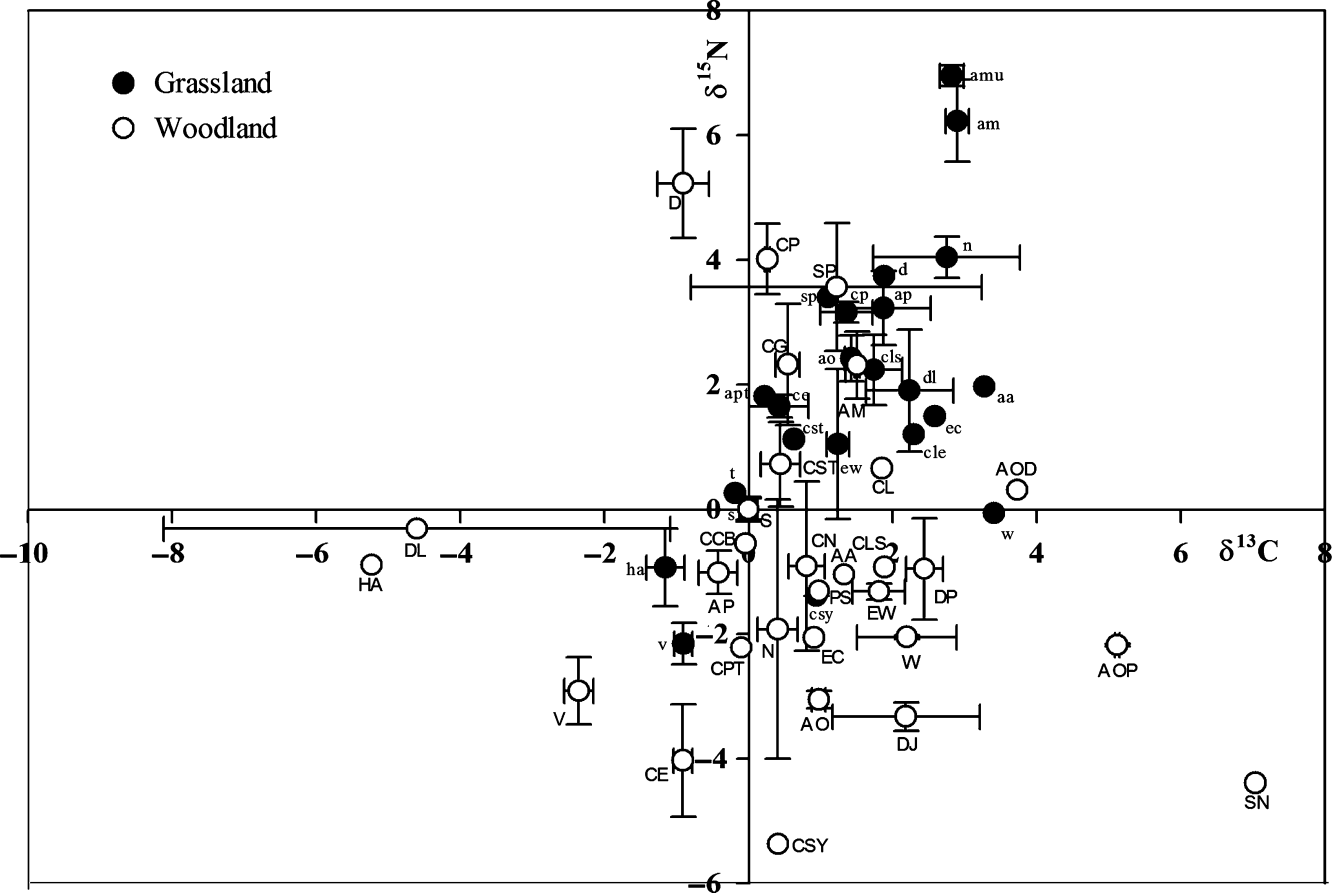
Isotopic composition of soil fauna within a grassland (black circles; lowercase labels) and a woodland (open circles; uppercase labels) habitats, with the soil stable isotope signature for each habitat set to zero and all the other results calibrated accordingly. Data presented as mean ± standard error, *n* = 3. s = soil for all other label codes see Table 2.

Data presented as mean ± standard error (*n* = 3), and *F*-values of a single-factor ANOVA *P < 0.05; **P < 0.001 indicating significant differences between habitats (df_1,4_ unless otherwise stated). Includes abbreviations used in Fig. 1.

To distinguish whether variation in signatures was due to habitat isotopic differences, the soil isotope values were set to zero in each habitat and the other results were normalized to account for this (sensu Erdmann et al. (2007)), (Fig. 1). Both *δ*^13^C and *δ*^15^N were significantly greater in the grassland soil compared with the woodland (*δ*^13^C *F*_1,66_ = 10.97; *P* = 0.002 and *δ*^15^N *F*_1,66_ = 16.55; *P* < 0.001), although tended to separate only on *δ*^15^N values. There were significant differences in delta signatures between fauna present in both habitats after calibration (Table S3). Taxa with significantly higher *δ*^13^C values in the grassland were the Mesostigmata (*F*_1,2_ = 46.83; *P* = 0.021), Diptera (*F*_1,2_ = 188.15; *P* = 0.005), and aphids (*F*_1,2_ = 81.49; *P* = 0.012), suggesting different C sources within the two habitats. While taxa with significantly higher *δ*^15^N values in the grassland, where the Oribatida (*F*_1,2_ = 599.74; *P* = 0.002), Prostigmata (*F*_1,2_ = 42.92; *P* = 0.023), Staphylinidae larvae (*F*_1,2_ = 234.10; *P* = 0.004), Entomobryomorpha (*F*_1,2_ = 528.50; *P* = 0.002), and woodlice (*F*_1,2_ = 3639.84; *P* < 0.001) (Table S3), suggesting the same fauna are at different trophic levels in the two habitats. Distinguishing between the differences in *δ*^15^N signatures, there are potentially different numbers of trophic levels in the two habitats. In the grassland, there appear to be only three trophic levels (*sensu* DeNiro and Epstein (1981)), one below soil (set to zero) and two above. While in the woodland, there appears to be four trophic levels, two with values lower than soil (zero) and two above.

Using the differences in stable isotopes across the whole soil fauna community (Layman et al. 2007), differences between habitats can be portrayed. The *δ*^15^N range (NR) of the soil fauna varied between the two habitats although not significantly, with a greater NR in the woodland (Table S4). The *δ*^13^C range (CR), however, was significantly greater in the woodland (*F*_1,4_ = 92.94; *P* < 0.001). The woodland fauna's isotopic signatures total area (TA) covered a significantly wider area (*F*_1,4_ = 94.78; *P* < 0.001; Table S4). The mean distance to centroid (CD) (a measure of trophic diversity within the web) was also significantly greater in the woodland (*F*_1,4_ = 103.2; *P* < 0.001; Table S4). The mean nearest neighbor distance (MNND) (a measure of the density of packing within an ecosystem) in the woodland was significantly greater than the grassland (*F*_1,4_ = 28.42; *P* = 0.006). The standard deviation of the nearest neighbor distance (SDNND) (a measure of the evenness of species packing) was significantly lower in the grassland than the woodland (*F*_1,4_ = 18.74; *P* = 0.012; Table S4) suggesting greater evenness.

We also wanted to test whether there was a difference between habitats when the organisms within the ecosystems where grouped by functionality rather than taxonomy. Using literature classifications, the invertebrate *δ*^13^C and *δ*^15^N results were consolidated into previously defined “feeding guilds” (Hunt et al. 1987; Hopkin 1997; Halaj et al. 2005; Krantz and Walter 2009) (Table 3) and the differences between the *δ*^13^C and *δ*^15^N signatures of these feeding guilds were assessed. For the majority of feeding guilds, there was no difference between the number of organisms found within each habitat (Table 3), only herbivores had a significantly greater number of individuals found in the grassland in comparison with the woodland (*F*_1,4_ = 15.80; *P* = 0.016); therefore, for the majority of feeding guilds, the differences in habitat cannot be attributed to a few organisms biasing the overall average at this taxonomic resolution.

**Table 3:** Groupings of invertebrates used for feeding guild analysis, includes average number of organisms (±SE) within each group per m^2^ in each habitat

Guild^1^	Organism	Grassland Number/m^2^	FPLSD *δ*^13^C	FPLSD *δ*^15^N	Woodland Number/m^2^	FLSD *δ*^13^C	FPLSD *δ*^15^N	Wardle Index
Herbivores*	Hemiptera: Aphidoidea							
	Coleoptera Larvae: Elateridae							
	Collembola: Symphypleona	2462 (±261)	a	a	1422 (±21)	ab	a	0.261 (±0.0467)
	Snails, Thrips							
Detritivores	Acari: Oribatida							
	Coleoptera: Ptiliidae							
	Diplopoda: Julidae/Polydesmidae	17443 (±5229)	b	b	17889 (±605)	b	a	−0.068 (±0.1775)
	Diptera Larvae, Earthworms							
	Enchytraeids, Woodlice							
Microbivores	Collembola: Entomobryomorpha	15958 (±2954)	a	b	12754 (±2337)	a	ab	0.114 (±0.0601)
	Collembola: Poduromorpha							
Omnivores	Acari: Astigmata							
	Acari: Prostigmata, Diptera	11841 (±3471)	b	b	17974 (±633)	ab	b	−0.245 (±0.1601)
Micro-predators	Acari: Mesostigmata							
	Coleoptera Larvae	4393 (±898)	b	c	4117 (±532)	b	b	0.016 (±0.0496)
	Pseudoscorpion							
Macro-predators	Chilopoda: Geophilomorpha							
	Coleoptera: Carabidae							
	Coleoptera: Staphylinidae	64 (±37)	ab	b	531 (±202)	ab	b	−0.822 (±0.1176)
	Spider							

Wardle Index Wardle (1995) estimating the stimulation (positive) or inhibition (negative) effect of agricultural grassland on soil fauna abundance.

* *P* < 0.05; indicating significant differences between habitats from single-factor ANOVA df_1,4_, combined with Fisher's protected least significant difference (FPLSD) test, different letters indicate significant differences between guilds.

1 Groupings ordered according to literature Hopkin (1997), Hunt et al. (1987), Halaj et al. (2005), Krantz and Walter (2009).

However, there may be relative differences in the response of the functional groups to the change from agricultural grassland to woodland. Prior to the stable isotope analysis of functional groups, an assessment of the variation in abundances was performed using an equation based on Wardle (1995). Herbivores had the most positive value of the index (Table 3), indicating that their abundance was the most stimulated by agriculture (in agreement with the above comparison of abundance), potentially due to the greater amounts of roots/living plant material in close proximity to the soil. Microbivores and micropredators also had positive values (Table 3), intimating their stimulation in the grassland in comparison with the woodland. Detritivores and omnivore functional groups had negative values alluding to their abundances being inhibited by the grassland, potentially due to greater amounts of detritus in the woodland (Table 3). The macropredator functional group result showed extreme inhibition in abundance in the grassland, compared with the woodland using the Wardle (1995) equation (Table 3) conceivably due to the greater litter and porosity in the woodland increasing the habitat capacity for these mobile predators.

Using stable isotope analysis to understand the differences between these functional groups, we found there was a significant difference between the *δ*^13^C and *δ*^15^N for the grouped feeding guilds for both *δ*^13^C (*F*_5,107_ = 2.77; *P* = 0.022) and *δ*^15^N (*F*_5,107_ = 13.12; *P* < 0.001). There were significant differences between habitat and feeding guild for both *δ*^13^C (habitat: *F*_1,101_ = 5.57; *P* = 0.020; guild: *F*_5,101_ = 2.43; *P* = 0.040) and *δ*^15^N (habitat: *F*_1,101_ = 4.98; *P* = 0.028; guild: *F*_5,101_ = 13.41; *P* < 0.001). However, the interaction between habitat and feeding guild was not significant for either *δ*^13^C or *δ*^15^N, suggesting that similar effects were occurring. There were significant differences in both *δ*^13^C and *δ*^15^N in the grassland for the different feeding guilds (*δ*^13^C *F*_5,41_ = 5.69 *P* < 0.001; *δ*^15^N *F*_5,41_ = 9.98 *P* < 0.001), while the woodland was only significantly different between feeding guilds for *δ*^15^N (*δ*^13^C *F*_5,60_ = 1.12 *P* = 0.362; *δ*^15^N *F*_5,60_ = 6.60 *P* < 0.001).

**Figure 2:**
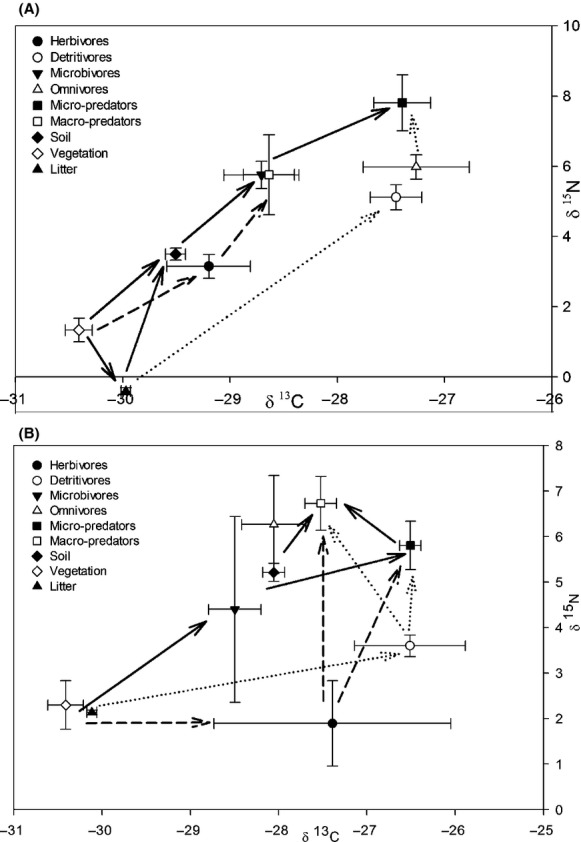
Isotopic composition of the grouped “trophic levels” for the (A) grassland habitat and (B) woodland habitat, average *δ*^13^C and *δ*^15^N (±standard error, *n* ≥ 6). See Table 3 for taxa included in each feeding group. Arrows representing different feeding pathways – solid microbial, dashed herbivory, and dotted detritivore.

The different energy pathways occurring are particularly distinctive in the grassland (Fig. 2A) where there appears to be three pathways, a detrital (or primary decomposer) pathway, an herbivory pathway, and a microbivorous (or secondary decomposer) pathway. This is very similar to the conceptual model described by Scheu (2002). For *δ*^13^C in the grassland, the herbivores and microbivores are significantly different to the detritivores, omnivores, and micropredators, while the macropredators are not significantly different to any of the other groups (ANOVA *F*_4,87_ = 7.99 *P* < 0.001; Table 3 FPLSD). Micropredators had significantly higher *δ*^15^N in the grassland soil, indicating that they are the top predator (Fig. 2A; ANOVA *F*_4,87_ = 9.52 *P* < 0.001; Table 3 FPLSD),whereas the *δ*^15^N of the macropredators suggests their main food source are herbivorous fauna. The detritivores, microbivores, and omnivores have very similar mean *δ*^15^N signatures, indicating a continuum of decomposition and predation.

In the woodland habitat, the postulated feeding channels are not as clearly defined as they were in the grassland (Fig. 2B). The *δ*^13^C signatures of the micropredators and detritivores were significantly different to the microbivores (ANOVA *F*_4,35_ = 5.74 *P* = 0.001; Table 3 FLSD), with the rest of the feeding guilds being similar to both, suggesting that the main food sources of micropredators are detritivores, as *δ*^13^C is food source specific. Two clusters appear through the analysis of *δ*^15^N signatures, one group includes the herbivores and detritivores at a significantly similar trophic level, compared with micro- and macropredators and microbivores, which cluster together at a similar trophic level (ANOVA *F*_4,35_ = 16.57 *P* < 0.001; Table 3 FPLSD). This indicates that herbivores and detritivores are the main prey of micropredators, while the microbivores are more likely to be predated by the macropredators (Fig. 2B) (agreeing with the *δ*^13^C results).

## Discussion

Our experiment, based on long-term research sites, has shown empirically clear differences between two different habitats on the same soil type, using stable isotope ratios as a proxy for the invertebrates trophic niche (Fig. 1). Twenty-five years prior to this study, the two habitats were both grassland and a change in management created the woodland. We found differences in functionality due to the different C inputs. The faunal communities dwelling within each habitat are of similar taxa but have altered food webs based on different basal resources, one driven by root derived resources, while the other appears to be litter derived. There were a greater number of predators occurring within the woodland habitat, and this may reflect the differences in plant diversity between the two habitats (Szanser et al. 2011). There was also a greater biomass of decomposer invertebrates within the woodland (e.g., Diplopoda: Polydesmidae and Oniscidea), possibly due in part to a greater amount of resources (Neher et al. 2012).

There were no grassland invertebrates with *δ*^15^N signatures lower than plant litter, while there were in the woodland (both Collembola: Symphypleona and snails), suggesting they may consume algae and lichens (Schneider et al. 2004; Tiunov 2007). Within the grassland habitat, there were few “litter” feeders, with the majority of organisms forming a continuum, with delta values greater than soil. In the woodland, a different SFW emerges with the majority of invertebrates clustering (and forming a continuum) from litter to soil. In the woodland, the macropredators are mainly Chilopoda, which can operate in the soil and litter layers, and have greater mobility, predating on the micropredators as well as the lower decomposer feeding guilds.

In general, *δ*^13^C does not fluctuate greatly between habitats due to minimal fractionation after consumption and assimilation and has been referred to as being “ecosystem specific”, (Peterson and Fry 1987). However, large differences in *δ*^13^C are found between organisms consuming different plant types (C_3_ or C_4_) (DeNiro and Epstein 1978). The two habitats appear to be relatively separated by their *δ*^15^N values (Fig. 1), although bulking of individual species within lineages may mask extremes, which could affect this level of separation. These results are similar to Hobson (1999) who separated two similar habitats by *δ*^15^N values of songbirds potentially consuming soil invertebrates in agricultural wetlands and boreal forests. Our results pose the tantalizing question of whether this level of isotopic separation of similar invertebrates in different habitats, but close locations, occurs regularly.

The main food sources of secondary decomposers are thought to be humified plant materials or the microbial community associated with plant litter and detritus (Hyodo et al. 2010). The isotopic signatures of secondary decomposers are usually enriched by 1–3‰ more than plant litter (Tiunov 2007). One taxa acting as a decomposer in the grassland but not in the woodland is the Poduromorpha, which were found to have high *δ*^15^N signatures in the woodland, suggesting they are microbivores, whereas in the grassland the Poduromorpha are located within the secondary decomposer boundary. Differences in fungal isotopic signatures (Kohzu et al. 1999) could be the reason why there is such a large variation in the isotope values of decomposers within habitats, and between habitats, rather than differences in trophic level.

Collembola are generally considered to be fungivorous; however, studies have found them to consume large amounts of bacteria (Murray et al. 2009; Crotty et al. 2011) and protozoa (Crotty et al. 2012). Subtle differences in the microbial community between habitats have the potential to affect the isotopic composition of a taxon, making it conceivable that they appear to be acting at different trophic levels dependent on habitat type. A study by Bonkowski et al. (2009) found the majority of soil invertebrates to be relying on C inputs from roots, breaking with the dogma that SFWs are fueled by plant litter inputs from above ground. Our results for the grassland suggest that the majority of soil fauna are utilizing sources other than litter; further investigation will confirm whether this is indeed a root driven food web.

There were only some significant differences between the soil fauna for C, N, and C/N ratio, between habitats, implying the fauna have a relatively constant body composition across space and feeding guild. Comparison of the C/N ratios in this study, to the seminal paper published by Hunt et al. (1987), highlights differences that may affect some of the many models and papers which have used this data (e.g., De Ruiter et al. 1993; Moore et al. 2005). Hunt's 1987 paper has been cited 329 times to date (according to the Web of Knowledge database accessed 1^st^ November 2013). The C/N ratios for all Acari were significantly different to those stated by Hunt et al. (1987), as were the Collembola in the grassland. Our results suggest that in Acari-dominated ecosystems, these large deviations from the ratios suggested by Hunt et al. (1987) could have greater effects than in Collembola dominated ecosystems. The Acari results were significantly different for both the woodland and grassland habitat, suggesting that habitat might not be a factor and this compositional difference is static between different habitats. It is unrealistic to consider organisms like the Acari with their hard exoskeleton (particularly the Mesostigmata, which had the lowest C/N ratio in comparison to Hunt) to have similar C/N ratios as soft-bodied taxa like the Collembola. The discrepancies between our empirical data and Hunt's could lead to a large knock-on effect when considering global C and N cycles, although these effects need further investigation.

Where the isotopic signatures of similar invertebrate orders are significantly different between habitats (Table 2 and Table S3), they may be utilizing different food sources or there may be differences in fractionation between the individual species within each group (Tiunov 2007). The Layman statistics (2007) were used to define how the two communities differ. The trophic length of the community does appear similar (NR). However, using the standard 3.4‰ amount to define trophic levels revealed a difference between the two habitats. Within an ecosystem, there is little variation between C isotopes when utilizing the same food source (≤5‰) (Staddon 2004). In the grassland, the *δ*^13^C range is ∼5‰, suggesting all the invertebrates are utilizing the same baseline food source. However, in the woodland CR, there is 12‰ difference, indicating a more complex food web. The woodland SFW appears to be based on more than one primary resource (Pollierer et al. 2009), providing for niche diversification at the base of the food web (Layman et al. 2007). The differences in *δ*^13^C signatures suggest that within this food web, there are soil feeders and litter feeders, as well as secondary decomposers.

The TA was wider in the woodland community, suggesting a greater trophic niche width and the aforementioned niche diversification. Habitat generalists usually have a wider trophic niche than organisms which are thought of as specialists (Coleman and Crossley 2003). In the conversion from grassland to woodland, it is likely that fauna were selected that are more generalist and can adapt to change. The CD is a function of species spacing (Layman et al. 2007) and is less affected by outliers (unlike TA), and in the woodland, the CD was significantly greater than the grassland, indicating that the woodland is more functionally diverse. The grassland taxa appear to have more functional redundancy (significantly smaller MNND) compared with the woodland and the SDNND is significantly smaller in the grassland suggesting a more even distribution of trophic niches.

Grouping fauna by functionality poststable isotope analysis allows us to understand the different pathways within the two habitats. Within the grassland, there appears to be defined feeding pathways visible (Fig. 2A), whereas these pathways are more ambiguous in the woodland. There were differences in the “top predator” between habitats, with the micropredators occupying the top position in the grassland, this agrees with a study focusing on Mesostigmata (Klarner et al. 2013) that found their stable isotope signatures to be similar to the macrofauna. It is probable that the same organisms are utilizing different food sources in the different habitats – due to different basal resources or potentially the taxa act as more generalist feeders in the woodland compared to the grassland. There are no specific predator–prey relationships within the soil (Crotty et al. 2012), this is reflected in stable isotope analysis at natural abundance where there appears to be a continuum of decomposition and predation. There is a lack of steps between trophic levels, with a truly omnivorous diet leading to isotopic signatures having a preferred feeding type as opposed to a definitive one. Omnivory is thought to be prominent within the soil food web (Scheu and Falca 2000), likely to be owing to the uncertainty over food resources in time and space.

Focusing on the Wardle index, there were certain functional groups stimulated in the grassland, in the order herbivores > microbivores > micropredators, while other groups were inhibited macropredators > omnivores > detritivores (Table 3). This emphasizes the likelihood that different basal resources are key to these differences between food webs. It also gives an indication that some groups may be switching function dependent on habitat. For example, omnivores are the detritivores in the grassland, whereas in the woodland, they are more akin to microbivores.

The difficulty within the study of SFWs is disentangling the different individual feeding preferences. Here, the trophic levels can be seen, but the full number of linkages is still dependent on species. An estimate of the number of trophic links within each food web (comparing the number of different guilds (Table S2) with hypothesized trophic links), our results agreed generally with Polis (1991) rather than Hunt et al. (1987).

There is a gap in the current understanding of stable isotope ecology linking the relationship between individual species and trophic level variation, with the connectivity of food webs (Vanderklift and Ponsard 2003). There is still limited agreement about how much fractionation occurs per trophic level for *δ*^15^N within the SFW. Historically, it was assumed to be 3.4‰ (DeNiro and Epstein 1981), but recent studies suggest that it is closer to 2‰ (McCutchan et al. 2003), particularly when analyzing the food web in the field (Illig et al. 2005). It is likely this difference in isotope values across trophic levels within the SFW is due to the mixing of food within the environment, with all “waste” being utilized by other organisms (coprophagy), and intraguild predation or carrion consumption increasing the potential for mixing the isotopic signatures. Furthermore, indirect consumption of microbial communities living on litter or fecal pellets may reduce the distinctiveness of trophic levels within the soil system.

## Conclusions

The results from this community assessment have shown differences between the two habitats, in invertebrate numbers, biomass, and stable isotope signatures. We have up to date C/N ratios compared with the literature, providing an alternative with the potential to begin to revise and modernize global C and N cycling models. Soil biota are known to play pivotal roles in biogeochemical processes; however, there is limited understanding in the global patterns of community structure (Fierer et al. 2009). This article demonstrates how differences in functionality are due to a variance in C inputs, with similar taxa utilizing different basal resources. Originally, the SFWs were identical, but due to a change in management and the conversion of a grassland to a woodland, different drivers have promoted a food web orientated toward root C in one habitat and litter C in the other.

## Supporting Information

**Table S1.** Soil and vegetation characteristics of the two experimental sites.

**Table S2**. Community composition, abundance and biomass (dry weight, mg^#^) of the macro- and mesofauna taxa from a grassland and woodland habitat.

**Table S3.** *F*-values of a single-factor ANOVA indicating significant differences between habitats for stable isotope signatures (all df_1,2_, apart from vegetation which was either df_1,8_ for live plant material, or df_1,10_ for dead plant material).

**Table S4.** Community-wide metrics (mean ± standard error [*n* = 6]), *F*-values of a single-factor ANOVA (df_1,2_) to assess the differences between the communities as a whole (analyzed through SIBER (Jackson et al. 2011) using Layman et al. (2007) metrics).
