# Supplementary material for: Divergence of feeding channels within the soil food web determined by ecosystem type
Source: Ecol Evol. 2013 Dec 4;4(1):1–13. doi: 10.1002/ece3.905 (PMC3894882; doi:10.1002/ece3.905)
Supplement: Supplementary file 1 [file ece30004-0001-SD1.docx]

**Table S.1: Soil and vegetation characteristics of the two experimental sites. *F*-values of a single factor ANOVA indicating significant differences between habitats (all df_1,10_, apart from live vegetation which was df_1,8_).**

|  | **Grassland** | **Woodland** | ***F-value*** | ***P-value*** |
| --- | --- | --- | --- | --- |
| **Soil** |  |  |  |  |
| **%C content** | 6.6 % (± 0.21) | 4.9 % (± 0.21) | 36.81 | <0.001 |
| **%N content** | 0.7 % (± 0.02) | 0.5 % (± 0.01) | 82.21 | <0.001 |
| **C:N ratio** | 10.1 (± 0.15) | 10.3 (± 0.21) | 0.65 | 0.440 |
| **Bulk Density** | 1.19 (± 0.063) | 1.22 (± 0.103) | 4.30 | 0.065 |
| **δ^13^C** | -29.5 ‰ (± 0.09) | -28.1 ‰ (± 0.13) | 86.10 | <0.001 |
| **δ^15^N** | 3.5 ‰ (± 0.17) | 5.2 ‰ (± 0.20) | 43.09 | <0.001 |
| **Vegetation** |  |  |  |  |
| **%C content** | 39.9 % (± 0.56) | 32.6 % (± 2.26) | 9.82 | 0.014 |
| **%N content** | 2.0 % (± 0.07) | 1.4% (± 0.07) | 35.50 | <0.001 |
| **C:N ratio** | 20.1 (± 0.68) | 23.1 (± 1.79) | 2.42 | 0.159 |
| **δ^13^C** | -30.4 ‰ (± 0.13) | -30.4 ‰ (± 0.20) | 0.01 | 0.990 |
| **δ^15^N** | 1.3 ‰ (± 0.33) | 2.3 ‰ (± 0.54) | 2.33 | 0.166 |
| **Litter** |  |  |  |  |
| **%C content** | 42.9 % (± 0.24) | 50.8 % (± 0.15) | 778.82 | <0.001 |
| **%N content** | 1.5 % (± 0.02) | 1.8% (± 0.03) | 102.23 | <0.001 |
| **C:N ratio** | 29.2 (± 0.33) | 28.7 (± 0.45) | 0.82 | 0.386 |
| **δ^13^C** | -30.0 ‰ (± 0.04) | -30.1 ‰ (± 0.06) | 4.14 | 0.069 |
| **δ^15^N** | -0.4 ‰ (± 0.10) | 2.1 ‰ (± 0.05) | 563.02 | <0.001 |

**Table S.2: Community composition, abundance and biomass (dry weight, mg^#^) of the macro- and mesofauna taxa from a grassland and woodland habitat. Data presented as mean ± standard error (n = 6), and *F*-values of a single factor ANOVA, * *P* < 0.05; ** *P* < 0.001 indicating significant differences between habitats (df_1,4_). ^#^ data presented as g for Earthworms due to biomass per m^2^ being an order of magnitude greater, even with gut content removed.**

|  | **Number per m^2^** | |  | **Biomass per m^2^ (mg)** | |  |
| --- | --- | --- | --- | --- | --- | --- |
|  | **Grassland** | **Woodland** | ***F-values*** | **Grassland** | **Woodland** | ***F-values*** |
| Acari: Astigmata | 1082 | 1167 (± 21.2) | 17.40* | 3.2 | 3.3 | 0.00 |
| Acari: Mesostigmata | 3204 (± 341.5) | 3883 (± 510.6) | 1.04 | 37.7 (± 7.27) | 77.5 (± 13.96) | 7.55 |
| Acari: Mesostigmata: Uropodidae | 934 (± 479.7) | 0 | 4.00 | 13.9 (± 7.00) | 0 | 3.99 |
| Acari: Oribatida | 16913 (± 5254.4) | 13475 (± 1118.7) | 0.05 | 54.5 (± 18.45) | 73.2 (± 8.92) | 0.88 |
| Acari: Oribatida: Damaeidae | 0 | 170 (± 21.2) | 4.00 | 0 | 21.6 | 4.00 |
| Acari: Oribatida: Phthiracaridae | 0 | 1231 (± 275.9) | 1156.76** | 0 | 66.8 (± 16.78) | 326.03** |
| Acari: Prostigmata | 10674 (± 3427.2) | 16510 (± 693.8) | 1.83 | 9.0 (± 4.04) | 12.6 (± 2.37) | 0.86 |
| Aphids (Hemiptera: Aphidoidea) | 700 (± 287.1) | 42 (± 42.4) | 6.86 | 34.4 (± 5.47) | 10.5 (± 10.53) | 4.12 |
| Chilopoda: Geophilomorpha | 0 | 85 (± 21.2) | 370.52** | 0 | 171.2 (± 75.56) | 65.35** |
| Coleoptera Larvae | 64 (± 36.8) | 106 (± 21.2) | 1.11 | 23.9 (± 11.93) | 14.6 (± 2.91) | 0.06 |
| Coleoptera Larvae: Elateridae | 85 (± 42.4) | 0 | 4.00 | 6.9 (± 3.47) | 0 | 4.00 |
| Coleoptera Larvae: Staphylinidae | 191 (± 127.3) | 85 (± 84.9) | 2.30 | 90.6 (± 7.94) | 21.1 (± 21.05) | 5.05 |
| Coleoptera: Carabidae | 0 | 64 (± 36.8) | 3.93 | 0 | 6.9 (± 3.97) | 3.80 |
| Coleoptera: Ptilidae | 0 | 127 (± 63.7) | 4.00 | 0 | 5.8 (± 2.88) | 4.00 |
| Coleoptera: Staphylinidae | 42 (± 42.4) | 212 (± 76.5) | 4.37 | 19.9 (± 19.88) | 201.2 (± 82.97) | 6.06 |
| Collembola: Entomobryomorpha | 13284 (± 2717.5) | 9401 (± 720.6) | 2.25 | 33.9 (± 2.7) | 21.8 (± 6.37) | 2.28 |
| Collembola: Neelipleona | 0 | 11226 (± 5684.6) | 4.00 | 0 | 7.2 (± 4.45) | 3.69 |
| Collembola: Poduromorpha | 2674 (± 1508.3) | 3353 (± 1724.5) | 0.00 | 4.9 (± 2.99) | 17 (± 8.5) | 0.39 |
| Collembola: Symphypleona | 1061 (± 21.2) | 1358 (± 21.2) | 94.05** | 1.4 | 2.9 | 0.00 |
| Diplopoda: Julidae | 0 | 42 (± 21.2) | 4.00 | 0 | 315.7 (± 234.03) | 3.82 |
| Diplopoda: Polydesmidae | 0 | 276 (± 148.5) | 114.96** | 0 | 169.3 (± 53.22) | 145.90** |
| Diptera | 42 (± 42.4) | 297 (± 112.3) | 5.55 | 1.5 (± 1.51) | 21.3 (± 6.91) | 14.62* |
| Diptera Larvae | 403 (± 76.5) | 255 (± 160.2) | 1.51 | 317.6 (± 190.67) | 4.0 (± 1.3) | 11.79* |
| Earthworm^#^ | 42 (± 21.2) | 64 | 1.00 | 1.2 g (± 0.49) | 1.4 g (± 0.11) | 0.72 |
| Enchytraeid worms | 64 (± 0) | 42 (± 42.4) | 2.49 | 12.5 (± 0) | 2.8 (± 2.82) | 6.14 |
| Pseudoscorpion | 0 | 42 (± 21.2) | 4.00 | 0 | 6.5 (± 3.25) | 4.00 |
| Snail | 0 | 21 (± 21.2) | 1.00 | 0 | 3.6 (± 3.57) | 1.00 |
| Spider | 21 (± 21.2) | 170 (± 139.2) | 0.80 | 8.9 (± 8.93) | 16.9 (± 8.57) | 0.47 |
| Thrips | 615 (± 21.2) | 0 | 33555** | 6.3 (± 0) | 0 | 0.00 |
| Woodlice | 21 (± 21.2) | 2207 (± 424.4) | 20.06* | 2.7 (± 2.72) | 208.6 (± 51.67) | 33.65* |

**Table S.2 continued: Community composition, abundance and biomass of the macro- and mesofauna taxa from a grassland and woodland habitat.**

**Table S.3: *F*-values of a single factor ANOVA indicating significant differences between habitats for stable isotope signatures (all df_1,2_, apart from vegetation which was either df_1,8_ for live plant material, or df_1,10_ for dead plant material). Stable isotope signature were normalised by setting the signatures of soil for each habitat to zero and all the other results were calibrated accordingly. Where there are no values the sample size was too small to perform the test, when the invertebrates were not present – np-g = not present in the grassland habitat; np-w = not present in the woodland habitat.**

| **Fauna** | **δ^13^C** | | **δ^15^N** | |
| --- | --- | --- | --- | --- |
|  | ***F-value*** | ***P-value*** | ***F-value*** | ***P-value*** |
| Acari: Astigmata |  |  |  |  |
| Acari: Mesostigmata | 46.83 | 0.021 | 16.11 | 0.057 |
| Acari: Mesostigmata: Uropodidae | np-w |  | np-w |  |
| Acari: Oribatida | 11.03 | 0.080 | 599.74 | 0.002 |
| Acari: Oribatida: Damaeidae | np-g |  | np-g |  |
| Acari: Oribatida: Phthiracaridae | np-g |  | np-g |  |
| Acari: Prostigmata | 4.19 | 0.177 | 42.92 | 0.023 |
| Aphids (Hemiptera: Aphidoidea) | 81.49 | 0.012 | 0.04 | 0.859 |
| Chilopoda: Geophilomorpha | np-g |  | np-g |  |
| Coleoptera Larvae |  |  |  |  |
| Coleoptera Larvae: Elateridae | np-w |  | np-w |  |
| Coleoptera Larvae: Staphylinidae | 5.73 | 0.139 | 234.10 | 0.004 |
| Coleoptera: Carabidae | np-g |  | np-g |  |
| Coleoptera: Ptilidae | np-g |  | np-g |  |
| Coleoptera: Staphylinidae | 0.16 | 0.727 | 0.00 | 0.978 |
| Collembola: Entomobryomorpha | 5.57 | 0.142 | 528.50 | 0.002 |
| Collembola: Neelipleona |  |  |  |  |
| Collembola: Poduromorpha | 6.13 | 0.132 | 1.41 | 0.357 |
| Collembola: Symphypleona |  |  |  |  |
| Diplopoda: Julidae | np-g |  | np-g |  |
| Diplopoda: Polydesmidae | np-g |  | np-g |  |
| Diptera | 188.15 | 0.005 | 13.94 | 0.065 |
| Diptera Larvae | 1.10 | 0.404 | 3.85 | 0.189 |
| Earthworm | 14.08 | 0.064 | 2.38 | 0.263 |
| Enchytraeid worms |  |  |  |  |
| Herbage | 7.67 | 0.024 | 0.22 | 0.654 |
| Litter | 504.81 | <0.001 | 59.82 | <0.001 |
| Nematodes | 4.35 | 0.172 | 8.77 | 0.098 |
| Pseudoscorpion | np-g |  | np-g |  |
| Snail |  |  |  |  |
| Spider | 0.04 | 0.859 | 0.02 | 0.891 |
| Thrips |  |  |  |  |
| Woodlice | 2.04 | 0.289 | 3639.84 | <0.001 |

**Table S.3 continued: *F*-values of a single factor ANOVA indicating significant differences between habitats.**

**Table S.4: Community wide metrics (mean ± standard error (n = 6)), *F*-values of a single factor ANOVA (df_1,2_) to assess the differences between the communities as a whole (analysed through SIBER (Jackson *et al.* 2011) using Layman (2007) metrics.**

| **Metrics** | **Grassland** | **Woodland** | ***F-value*** | ***P-value*** |
| --- | --- | --- | --- | --- |
|  |  |  |  |  |
| δ^15^N range (NR) | 8.64 ‰ (± 0.268) | 10.74 ‰ (± 0.771) | 6.62 | 0.062 |
| δ^13^C range (CR) | 4.56 ‰ (± 0.265) | 12.20 ‰ (± 0.963) | 92.94 | <0.001 |
| Total area convex hull (TA) | 19.4 (± 1.02) | 79.0 (± 6.03) | 94.78 | <0.001 |
| Mean distance to centroid (CD) | 1.99 (± 0.085) | 2.96 (± 0.043) | 103.2 | <0.001 |
| Mean nearest neighbour distance (MNND) | 0.75 (± 0.047) | 1.11 (± 0.047) | 28.42 | 0.006 |
| Standard deviation of nearest neighbour distance (SDNND) | 0.56 (± 0.061) | 0.97 (± 0.071) | 18.74 | 0.012 |
